# Comparing the sensitivities of two screening tests in nonblinded randomized paired screen‐positive trials with differential screening uptake

**DOI:** 10.1002/sim.9215

**Published:** 2021-10-10

**Authors:** Peter M. van de Ven, Andrea Bassi, Johannes Berkhof

**Affiliations:** ^1^ Department of Epidemiology and Data Science Amsterdam UMC, Vrije Universiteit Amsterdam Amsterdam The Netherlands

**Keywords:** differential uptake, nonblinded, randomized controlled trials, screening, sensitivity

## Abstract

Before a new screening test can be used in routine screening, its performance needs to be compared to the standard screening test. This comparison is generally done in population screening trials with a screen‐positive design where participants undergo one or both screening tests after which disease verification takes place for those positive on at least one screening test. We consider the randomized paired screen‐positive design of Alonzo and Kittelson where participants are randomized to receive one of the two screening tests and only participants with a positive screening test subsequently receive the other screening test followed by disease verification. The tests are usually offered in an unblinded fashion in which case the screening uptake may differ between arms, in particular when one test is more burdensome than the other. When uptake is associated with disease, the estimator for the relative sensitivity derived by Alonzo and Kittelson may be biased and the type I error of the associated statistical test is no longer guaranteed to be controlled. We present methods for comparing sensitivities of screening tests in randomized paired screen‐positive trials that are robust to differential screening uptake. In a simulation study, we show that our methods adequately control the type I error when screening uptake is associated with disease. We apply the developed methods to data from the IMPROVE trial, a nonblinded cervical cancer screening trial comparing the accuracy of HPV testing on self‐collected versus provider‐collected samples. In this trial, screening uptake was higher among participants randomized to self‐collection.

## 1 INTRODUCTION

Screening is an important aspect of preventive healthcare with many countries nowadays having population screening programs implemented for several diseases. Population screening programs aim to detect disease in an early asymptomatic phase in order to facilitate timely treatment. Before a new screening test can be used in routine screening, its performance needs to be compared to the standard screening test. Important measures of the performance of a screening test are the sensitivity and the false positive fraction, the latter also being referred to as 1‐specificity. The sensitivity is the proportion of diseased subjects in the population with a positive screening test and the false positive fraction is the proportion of nondiseased subjects in the population with a positive screening test. A good screening test has both a high sensitivity and a low false positive fraction.

Pepe^1^ categorizes the development of a medical test into five phases of research. At the end of the fourth phase, the threshold for defining test positivity has been determined and estimates of the sensitivity and specificity have been obtained. The fifth and final phase of research consists of an assessment of the impact of the test in real life. In the setting of population screening, this is generally done in large population screening trials where subjects in the intended screening population are randomized to either receive the new screening test or the standard screening test. Subjects who are positive on the screening test subsequently receive a gold standard test for disease verification as part of the diagnostic work‐up. Subjects who are negative on the screening test do not undergo disease verification as most gold standard tests are invasive and costly and the risk of having the disease is considered to be low after a negative screening test.

The above design is generally referred to as an unpaired screen‐positive design.^1^ Since true disease status is only verified for subjects positive on the screening test, absolute sensitivity and specificity cannot be estimated. Schatzkin et al,^2^ however, showed that estimation of the relative sensitivity and relative false positive fraction is still possible if the underlying disease prevalence does not differ between the two arms. By way of illustration, suppose that the number of subjects is equal in the two arms. Then, by Schatzkin et al,^2^ the ratio of the number of detected disease cases in the two arms is an estimate of the relative sensitivity and the ratio of the number of subjects with a false positive test in the two arms is an estimate of the relative false positive fraction.

Alonzo and Kittelson^3^ introduced the randomized paired screen‐positive design. The design is similar to the unpaired screen‐positive design, except that subjects with a positive screening test also receive the other screening test in addition to the gold standard test. Alonzo and Kittelson^3^ derived estimators of the relative sensitivity and the relative false positive fraction in a randomized paired screen‐positive trial. Their estimators are unbiased under the assumption that outcomes of the screening tests and gold standard test are missing at random.

New screening tests are nowadays often developed to reduce the harms or anxiety associated with the sampling procedure and/or to increase the speed of the sampling procedure. Examples include blood tests, urine tests, home swab/brush tests and digital tests as alternatives to more invasive test procedures. A key feature of trials evaluating rapid, less invasive tests is that trial participants are unblinded with respect to the screening test. This may lead to unbalancedness because subjects who refused the standard screening test at previous invitations are expected to have a high drop‐out probability when randomized to the control arm. Those subjects are also at increased disease risk because screening nonattendance is known to be strongly associated with disease. In such cases, the assumptions underlying the estimators of Schatzkin et al^2^ and Alonzo and Kittelson^3^ are unlikely to hold.

An example of a randomized paired‐screen positive trial where blinding was not possible is the IMPROVE trial^4^ evaluating HPV testing on self‐collected samples in cervical cancer screening. The trial compared the new HPV test with HPV testing on a provider‐collected sample. The gold standard test was histologically confirmed high‐grade cervical intraepithelial neoplasia or cancer (CIN3+). Withdrawal from the study was found to be especially high among women randomized to receive the more burdensome provider‐collected test first. It cannot be ruled out those women who withdrew from the provider‐collection arm represent a subgroup of women with a poor screening history and hence an elevated CIN3+ risk. In that case, nonblinding has induced a difference in disease risk between the two trial arms causing the estimator of Alonzo and Kittelson to be biased.

In this article we present an alternative method for estimating the relative sensitivity and the relative false positive fraction in the randomized paired screen‐positive design. Like the randomized paired screen‐positive design, our method requires that patients positive on a screening test also receive the other screening test, but it allows for a difference in underlying disease prevalence between the two arms. After conditioning on the true disease status, Wald, score and likelihood ratio test procedures for the relative risk in two‐by‐two contingency tables can be applied to test noninferiority and superiority of the relative sensitivity and relative false positive fraction. Results of the first screening test are allowed to be missing not at random with missingness dependent on presence of the disease and the arm to which the subject is randomized. Results of the second screening test and the gold standard test following a positive first screening test are assumed to be missing at random.

The remainder of the article is organized as follows. In Section 2 we introduce the notation, describe the Alonzo and Kittelson method and present alternative statistical tests. In Section 3 we describe the design of a simulation study in which the empirical type I error and power of the tests are assessed. The results of this simulation study are summarized in Section 4. In Section 5 we reanalyze the data from the IMPROVE trial using the alternative methods developed. We conclude with a discussion in Section 6.

## 2 METHODS

We consider the setting in which the performance of a new screening test A is evaluated against the standard screening test B. We assume that a gold standard test exists that verifies the true disease status of the subjects. Let A+, B+, and D+ represent positive results on the new screening test A, the standard screening test B and the gold standard test, respectively. The sensitivities of the screening tests are ${\text{Sens}}_{A}=P\left(A\;+|D+\right)$ and ${\text{Sens}}_{B}=P\left(B\;+\;|D+\right)$. Let Δ denote the relative sensitivity ${\text{Sens}}_{A}/{\text{Sens}}_{B}$ and let $\delta_{0}$ be a prespecified lower margin for Δ. We consider testing of the hypothesis

(1) $$H_{0}:\Delta \leq \delta_{0}\;\text{against}\;H_{1}:\Delta >\delta_{0}.$$

Statement (1) is a noninferiority test hypothesis when $\delta_{0}<1$ and a superiority test hypothesis when $\delta_{0}=1$.

We consider a randomized paired‐screen positive design in which *M* subjects are enrolled. Let $M^{A}$ be the number of subjects who are randomized to the arm that first receives the new screening test. Let $M^{B}$ be the number of subjects who are randomized to the arm that first receives the standard screening test. Only subjects who are positive on the first screening test subsequently undergo the other screening test and the gold standard test. Follow‐up for a subject is said to be complete when the first screening test is negative or when the first screening test is positive and results of both the other screening test and the gold standard test have been obtained. Let $N^{A}$ and $N^{B}$ denote the number of subjects with complete follow‐up in each of the arms and let $N=N^{A}+N^{B}$.

Table 1 summarizes the data for the *N* screened subjects where we make a distinction between observed and unobserved cell counts. The number of subjects who are randomized to the arm that receives the new screening test first and complete follow‐up can be decomposed as $N^{A}=N_{DAB}^{A}+N_{DA\bar{B}}^{A}+N_{\bar{D}AB}^{A}+N_{\bar{D}A\bar{B}}^{A}+N_{\bar{A}}^{A}$. In this decomposition, $N_{DAB}^{A}$ denotes the number of subjects who first receive the new screening test and have positive test results on both screening tests and the gold standard test. $N_{DA\bar{B}}^{A}$ denotes the number of subjects who first receive the new screening test and have positive test results on the new screening test and gold standard test and a negative test result on the standard screening test. $N_{\bar{D}AB}^{A}$ denotes the number of subjects who first receive the new screening test and have positive test results on both screening tests and a negative test result on the gold standard test. $N_{\bar{D}A\bar{B}}^{A}$ denotes the number of subjects who first receive the new screening test and have a positive test result on the new screening test and negative test results on the standard screening test and gold standard test. $N_{\;\bar{A}}^{A}$ denotes the total number of subjects who first receive the new screening test and are negative. The number $N_{\;\bar{A}}^{A}$ is observed in the study, but by design it is unknown how the $N_{\;\bar{A}}^{A}$ subjects are distributed across categories defined by the standard screening test and the gold standard test. In the same way, the number of subjects who are randomized to the arm that receives the standard screening test first and complete follow‐up can be decomposed as $N^{B}=N_{DAB}^{B}+N_{\bar{D}AB}^{B}+N_{D\;\bar{A}B}^{B}+N_{\bar{D}\;\bar{A}B}^{B}+N_{\bar{B}}^{B}$. In this decomposition, $N_{DAB}^{B}$ denotes the number of subjects who first receive the standard screening test and have positive test results on both screening tests and the gold standard test. $N_{D\;\bar{A}B}^{B}$ denotes the number of subjects who first receive the standard screening test and have positive test results on the standard screening test and gold standard test and a negative test result on the new screening test. $N_{\bar{D}AB}^{B}$ denotes the number of subjects who first receive the standard screening test and have positive test results on both screening tests and a negative test result on the gold standard test. $N_{\bar{D}\;\bar{A}B}^{B}$ denotes the number of subjects who first receive the standard screening test and have a positive test result on the standard screening test and negative test results on the new screening test and gold standard test. $N_{\bar{B}}^{B}$ denotes the total number of subjects who first receive the standard screening test and are negative.

**TABLE 1:** Data from a randomized paired screen‐positive study comparing the sensitivity of screening tests A and B

	B+,D+	B‐,D+	Total D+	B+,D‐	B‐,D‐	Total D‐
$M^{A}$ randomized to receive new test; $N^{A}$ actually screened						
A+	$N_{DAB}^{A}$	$N_{DA\bar{B}}^{A}$	$N_{DA}^{A}$	$N_{\bar{D}AB}^{A}$	$N_{\bar{D}A\bar{B}}^{A}$	$N_{\bar{D}A}^{A}$
A‐	$\left[N_{D\;\bar{A}B}^{A}\right]$	$\left[N_{D\;\bar{A}\;\bar{B}}^{A}\right]$	$\left[N_{D\;\bar{A}}^{A}\right]$	$\left[N_{\bar{D}\;\bar{A}B}^{A}\right]$	$\left[N_{\bar{D}\;\bar{A}\;\bar{B}}^{A}\right]$	$\left[N_{\bar{D}\;\bar{A}}^{A}\right]$
Total	$\left[N_{DB}^{A}\right]$	$\left[N_{D\bar{B}}^{A}\right]$	$\left[N_{D}^{A}\right]$	$\left[N_{\bar{D}B}^{A}\right]$	$\left[N_{\bar{D}\;\bar{B}}^{A}\right]$	$\left[N_{\bar{D}}^{A}\right]$
$M^{B}$ randomized to receive standard test first; $N^{B}$ actually screened						
A+	$N_{DAB}^{B}$	$\left[N_{DA\bar{B}}^{B}\right]$	$\left[N_{DA}^{B}\right]$	$N_{\bar{D}AB}^{B}$	$\left[N_{\bar{D}A\bar{B}}^{B}\right]$	$\left[N_{\bar{D}A}^{B}\right]$
A‐	$N_{D\;\bar{A}B}^{B}$	$\left[N_{D\;\bar{A}\;\bar{B}}^{B}\right]$	$\left[N_{D\;\bar{A}}^{B}\right]$	$N_{\bar{D}\;\bar{A}B}^{B}$	$\left[N_{\bar{D}\;\bar{A}\;\bar{B}}^{B}\right]$	$\left[N_{\bar{D}\;\bar{A}}^{B}\right]$
Total	$N_{DB}^{B}$	$\left[N_{D\bar{B}}^{B}\right]$	$\left[N_{D}^{B}\right]$	$N_{\bar{D}B}^{B}$	$\left[N_{\bar{D}\;\bar{B}}^{B}\right]$	$\left[N_{\bar{D}}^{B}\right]$

*Note*: Cell counts in brackets are missing by design.

Under the assumption that the observed cell counts in each arm are realizations of independent multinomial distributions, the log‐likelihood for the observed data is proportional to

(2) $$\begin{array}{ll} \ell_{AK} & =N_{DAB}^{A}\log[P_{A}\left(D+,A+,B+\right)]\\ & \;+N_{DA\bar{B}}^{A}\log[P_{A}\left(D+,A+\right)-P_{A}\left(D+,A+,B+\right)]\\ & \;+N_{\bar{D}AB}^{A}\log[P_{A}\left(D-,A+,B+\right)]\\ & \;+N_{\bar{D}A\bar{B}}^{A}\log[P_{A}\left(D-,A+\right)-P_{A}\left(D-,A+,B+\right)]\\ & \;+N_{\;\bar{A}}^{A}\log[1-P_{A}\left(D+,A+\right)-P_{A}\left(D-,A+\right)]\\ & \;+N_{DAB}^{B}\log[P_{B}\left(D+,A+,B+\right)]\\ & \;+N_{D\;\bar{A}B}^{B}\log[P_{B}\left(D+,B+\right)-P_{B}\left(D+,A+,B+\right)]\\ & \;+N_{\bar{D}AB}^{B}\log[P_{B}\left(D-,A+,B+\right)]\\ & \;+N_{\bar{D}\;\bar{A}B}^{B}\log[P_{B}\left(D-,B+\right)-P_{B}\left(D-,A+,B+\right)]\\ & \;+N_{\bar{B}}^{B}\log[1-P_{B}\left(D+,B+\right)-P_{B}\left(D-,B+\right)], \end{array}$$

where $P_{A}\left(\cdot \right)$ and $P_{B}\left(\cdot \right)$ denote probabilities of combinations of test outcomes that are conditional on the subjects first receiving test A and B, respectively.

### 2.1 The Alonzo and Kittelson estimator for the relative sensitivity

When all the subjects in the study show up for screening, the probabilities in (2), that is, $P_{A}\left(\cdot \right)$ and $P_{B}\left(\cdot \right)$, can be assumed to equal the corresponding probabilities $P\left(\cdot \right)$ in the study population. The estimator for the relative sensitivity derived by Alonzo and Kittelson^3^ under the assumption that $P_{A}\left(\cdot \right)=P_{B}\left(\cdot \right)$ is

(3) $${\hat{\Delta }}_{AK}=\frac{N_{DAB}^{A}+N_{DAB}^{B}+N_{DA\bar{B}}^{A}/\hat{\gamma }}{N_{DAB}^{A}+N_{DAB}^{B}+N_{D\;\bar{A}B}^{B}/\left(1-\hat{\gamma }\right)},$$

where

(4) $$\hat{\gamma }=\frac{N^{A}-N_{AB}^{A}}{N-N_{AB}}.$$

$N_{AB}$ is the number of subjects with positive test results on both screening tests and $N_{AB}^{A}$ is the number of subjects with two positive screening test results who received the new screening test first. The two‐sided (1−2α) × 100% confidence interval for the logarithm of the relative sensitivity derived by Alonzo and Kittelson can be used for testing the noninferiority and superiority test hypothesis in statement (1) at a one‐sided significance level of α × 100%.

In case of differential screening uptake, the assumption that the probabilities $P_{A}\left(\cdot \right)$ and $P_{B}\left(\cdot \right)$ in Equation (2) are the same is flawed. Furthermore, $\hat{\gamma }$ defined in Equation (4) is assumed not to depend on disease status. However, it is used for weighting diseased subjects in Equation (3). This implies that the estimator in Equation (3) is biased when screening uptake is related to disease status.

### 2.2 A conditional estimator for the relative sensitivity

We derive an alternative estimator for the relative sensitivity that remains unbiased under differential screening uptake. Employing Bayes' theorem it follows that

$$P_{A}\left(A+\right)=\frac{P_{A}\left(A+,B+\right)}{P_{A}\left(B\;+|A+\right)}$$

and

$$P_{B}\left(B+\right)=\frac{P_{B}\left(A+,B+\right)}{P_{B}\left(A\;+|B+\right)}.$$

Conditioning these probabilities on D+ gives

$$P_{A}\left(A+|D+\right)=\frac{P_{A}\left(A+,B\;+|D+\right)}{P_{A}\left(B\;+|A+,D+\right)}$$

and

$$P_{B}\left(B+|D+\right)=\frac{P_{B}\left(A+,B\;+|D+\right)}{P_{B}\left(A\;+|B+,D+\right)}.$$

We assume that the sensitivity of screening test A in subjects receiving test A first is equal to the sensitivity of screening test A when administered to the whole study population. Thus, $P_{A}\left(A\;+|D+\right)=P\left(A\;+|D+\right)$. Similarly, we assume $P_{B}\left(B\;+|D+\right)=P\left(B\;+|D+\right)$. In addition, we assume that $P_{A}\left(A+,B+|D+\right)=P_{B}\left(A+,B\;+|D+\right)$, which means that the probability of being positive on both screening tests for the diseased subjects does not depend on the order of the tests. Then the relative sensitivity equals

$$\Delta =\frac{P\left(A\;+|D+\right)}{P\left(B\;+|D+\right)}=\frac{P_{B}\left(A\;+|B+,D+\right)}{P_{A}\left(B\;+|A+,D+\right)}.$$

From this we derive a conditional estimator for the relative sensitivity as

(5) $${\hat{\Delta }}_{C}=\frac{N_{DAB}^{B}/N_{DB}^{B}}{N_{DAB}^{A}/N_{DA}^{A}},$$

where $N_{DB}^{B}=N_{DAB}^{B}+N_{D\;\bar{A}B}^{B}$ and $N_{DA}^{A}=N_{DAB}^{A}+N_{DA\bar{B}}^{A}$. In the Supplementary Material, a similar estimator for the relative false positive fraction is derived by conditioning the probabilities on D−.

### 2.3 Statistical tests based on the conditional estimator

Since the numerator and denominator on the right side of Equation (5) are proportions from two independent binomial samples, we can use standard statistical tests for testing the noninferiority or superiority of the relative risk. For testing superiority (ie, $\delta_{0}=1$), the chi‐square test or Fisher's exact test can be used, whereas for noninferiority (ie, $\delta_{0}<1$), the statistical tests derived in Miettinen and Nurminen^5^ and Farrington and Manning^6^ can be used. For ease of notation we define $\pi_{A}=P_{B}\left(A\;+|B+,D+\right)$ and $\pi_{B}=P_{A}\left(B+|A+,D+\right)$ and reformulate hypothesis (1) as

(6) $$H_{0}:\frac{\pi_{A}}{\pi_{B}}\leq \delta_{0}\;\text{against}\;H_{1}:\frac{\pi_{A}}{\pi_{B}}>\delta_{0}.$$

The statistical tests that will be employed to test hypothesis (6) are the Wald, score and likelihood ratio test.

Let ${\hat{\pi }}_{A}=N_{DAB}^{B}/N_{DB}^{B}$ and ${\hat{\pi }}_{B}=N_{DAB}^{A}/N_{DA}^{A}$ denote the unrestricted maximum likelihood estimators of $\pi_{A}$ and $\pi_{B}$, respectively. The Wald statistic for testing hypothesis (6) is

$$T_{W}=\frac{{\hat{\pi }}_{A}-\delta_{0}\;{\hat{\pi }}_{B}}{\sqrt{\hat{\mathrm{Var}}\left({\hat{\pi }}_{A}-\delta_{0}\;{\hat{\pi }}_{B}\right)}},$$

where

$$\hat{\mathrm{Var}}\left({\hat{\pi }}_{A}-\delta_{0}\;{\hat{\pi }}_{B}\right)=\frac{{\hat{\pi }}_{A}\left(1-{\hat{\pi }}_{A}\right)}{N_{DB}^{B}}+\delta_{0}^{2}\;\frac{{\hat{\pi }}_{B}\left(1-{\hat{\pi }}_{B}\right)}{N_{DA}^{A}}.$$

Under the null hypothesis $T_{W}$ asymptotically follows a standard normal distribution.

A score test requires the maximum likelihood estimators for the parameters under the null hypothesis. The constrained maximum likelihood estimator of $\pi_{B}$ under the null constraint $\pi_{A}=\delta_{0}\;\pi_{B}$, derived in Miettinen and Nurminen,^5^ is

$${\tilde{\pi }}_{B}=\frac{-b-\sqrt{b^{2}-4ac}}{2a},$$

where $a=\delta_{0}\left(N_{DA}^{A}+N_{DB}^{B}\right)$, $b=-\left(\delta_{0}N_{DB}^{B}+N_{DAB}^{B}+N_{DA}^{A}+\delta_{0}N_{DAB}^{A}\right)$ and $c=N_{DAB}^{A}+N_{DAB}^{B}$. The constrained maximum likelihood estimator of $\pi_{A}$ is ${\tilde{\pi }}_{A}=\delta_{0}\;{\tilde{\pi }}_{B}$. A score‐type test statistic for testing hypothesis (6) is

$$T_{S}=\frac{{\hat{\pi }}_{A}-\delta_{0}{\hat{\pi }}_{B}}{\sqrt{\tilde{\mathrm{Var}}\left({\hat{\pi }}_{A}-\delta_{0}{\hat{\pi }}_{B}\right)}},$$

where

$$\tilde{\mathrm{Var}}\left({\hat{\pi }}_{A}-\delta_{0}\;{\hat{\pi }}_{B}\right)=\left(\frac{{\tilde{\pi }}_{A}\left(1-{\tilde{\pi }}_{A}\right)}{N_{DB}^{B}}+\delta_{0}^{2}\;\frac{{\tilde{\pi }}_{B}\left(1-{\tilde{\pi }}_{B}\right)}{N_{DA}^{A}}\right)\frac{N_{DA}^{A}+N_{DB}^{B}}{N_{DA}^{A}+N_{DB}^{B}-1}$$

as derived in Miettinen and Nurminen^5^ with the tilde denoting an estimator constrained by ${\tilde{\pi }}_{A}=\delta_{0}\;{\tilde{\pi }}_{B}$. Under the null hypothesis $T_{S}$ asymptotically follows a standard normal distribution.

To derive the likelihood ratio test statistic for testing hypothesis (6) we note that the log‐likelihood for the cell counts $N_{DAB}^{A}$, $N_{DA\bar{B}}^{A}$, $N_{DAB}^{B}$ and $N_{D\;\bar{A}B}^{B}$ after conditioning on $N_{DA}^{A}$ and $N_{DB}^{B}$ is proportional to

$$\ell_{C}\left(\pi_{A},\pi_{B}\right)=N_{DAB}^{B}\log\left(\pi_{A}\right)+N_{D\;\bar{A}B}^{B}\log\left(1-\pi_{A}\right)+N_{DAB}^{A}\log\left(\pi_{B}\right)+N_{DA\bar{B}}^{A}\log\left(1-\pi_{B}\right).$$

The likelihood ratio test statistic for testing hypothesis (6) is

$$T_{LR}=\left\{\begin{array}{ll} -2\left[\ell_{C}\left({\tilde{\pi }}_{A},{\tilde{\pi }}_{B}\right)-\ell_{C}\left({\hat{\pi }}_{A},{\hat{\pi }}_{B}\right)\right] & if\;{\hat{\pi }}_{A}-\delta_{0}\;{\hat{\pi }}_{B}\geq 0;\\ 0 & if\;{\hat{\pi }}_{A}-\delta_{0}\;{\hat{\pi }}_{B}<0. \end{array}\right.$$

Under the null hypothesis the asymptotic distribution of $T_{LR}$ is a mixture of chi‐square distributions with 0 and 1 degree of freedom: $\frac{1}{2}\chi_{0}^{2}+\frac{1}{2}\chi_{1}^{2}$.

## 3 SIMULATIONS

Simulation studies are performed to evaluate the empirical type I errors of the four different statistical tests in settings with perfect and settings with imperfect screening uptake. In the settings with perfect screening uptake, 0% withdrawal is assumed in both arms. In the settings with imperfect screening uptake, 0% withdrawal is assumed in the arm randomized to the new screening test A and 20% withdrawal is assumed in the arm randomized to the standard screening test B. In addition, power of the four different statistical tests is compared under perfect screening uptake.

The number of subjects randomized to each arm is assumed equal and set at 5000 and 10 000. The disease prevalence in subjects receiving the new test A first is set at 0.01, 0.05, and 0.10. In the arm with standard screening test B as first test, we set the disease prevalence in the subjects actually screened equal to 0.5, 0.75, 1, and 1.25 times the prevalence in the population randomized to the new test A first. The sensitivity of standard screening test B is set at 0.75, 0.85, and 0.95. We set the margin $\delta_{0}$ for the relative sensitivity at 0.90, 0.95, and 1. Specificities of test A and B are set at 0.90, 0.95, and 0.99. The odds ratio of new screening test A versus standard screening test B outcomes is set at 1, 2, and 5, both in subjects with and without the disease. The one‐sided significance level is set at 5%.

When evaluating the power, the relative sensitivity under the alternative is set at 1 for $\delta_{0}=0.90$ and $\delta_{0}=0.95$, and at 1.05 for $\delta_{0}=1$. In each setting, the number of subjects randomized is set such that the power for the Wald test based on the conditional estimator equals 80%. This is done by first calculating for each arm the required number of subjects with the disease who are positive on the first screening test using the sample size formula of Farrington and Manning.^6^ The total number of subjects to be randomized is derived from these numbers using estimates for the disease prevalence and the sensitivity of the standard screening test. Details and formula are provided in the Supplementary Material, where we also explain how the formula can be used to determine the sample size in settings where differential screening uptake is anticipated.

An additional simulation study is performed to evaluate the Alonzo and Kittelson estimator and conditional estimator with respect to bias, precision and coverage probabilities of 95% confidence intervals in settings with perfect and imperfect screening uptake. Details of this simulation study and its results are provided in the Supplementary Material, which also contains a detailed description of the simulation studies for evaluating empirical type I errors and power of the statistical tests. All simulations are performed in R. The simulation size is set at 10 000 replications per setting. In simulation runs where $N_{DAB}^{A}$, $N_{DAB}^{B}$, $N_{DA\bar{B}}^{A}$, and/or $N_{D\;\bar{A}B}^{B}$ are zero, we add 0.25 to each of these four counts.

## 4 RESULTS

**FIGURE 1:**
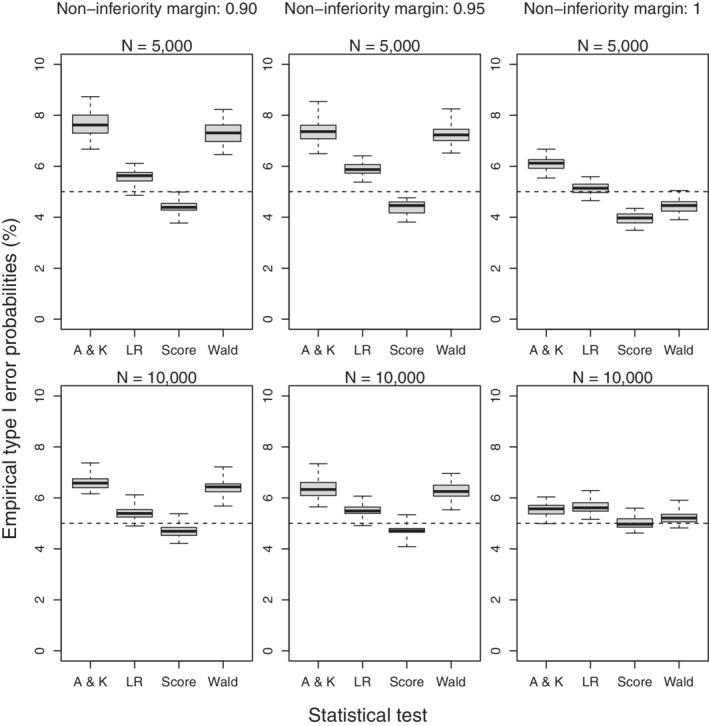
Boxplots of the empirical type I error probabilities of the Alonzo and Kittelson (A & K) test, likelihood ratio (LR) test, score test and Wald test in setting with perfect screening uptake. Disease prevalence is 0.01 and the sensitivity of the standard screening test is 0.95. Boxes represent quartiles and median and whiskers represent the minimum and maximum

**FIGURE 2:**
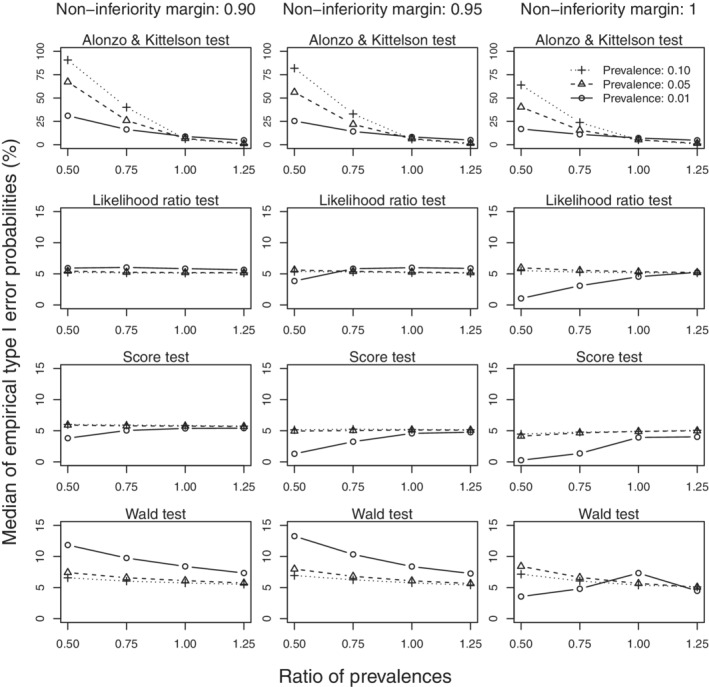
Median empirical type I error probabilities of the four tests in setting with 20% no show in the arm receiving the standard screening test first. Perfect screening uptake is assumed for the arm receiving the new screening test first. Ratios of prevalences refer to the disease prevalence in the subjects actually screened in the arm receiving the standard test first relative to the disease prevalence in the arm receiving the new test first. Prevalences 0.01, 0.05, and 0.10 refer to disease prevalences in the arm receiving the new test first. 5000 subjects are randomized to each arm and the sensitivity of standard screening test is set at 0.95

Figure 1 shows the empirical type I errors of the four different tests for the setting with perfect screening uptake where disease prevalence is 0.01 and the sensitivity of the standard screening tests is 0.95. In this setting, empirical type I errors for both the test based on the Alonzo and Kittelson estimator and the alternative Wald test proposed in this article exceed the nominal level, reaching 7.5% when 5000 subjects are randomized to each arm. The likelihood ratio test and the score test show adequate control of the type I error with the score test being slightly conservative. In settings with higher prevalence or lower sensitivity all four tests were found to adequately control the type I error. Figure 2 shows the empirical type I errors of the four different tests for the setting with 20% withdrawal in subjects randomized to the standard screening test first and where 5000 subjects are randomized to each arm and sensitivity of the standard test is 0.95. The empirical type I errors of the test based on the Alonzo and Kittelson estimator are not controlled when screening uptake is differential and disease‐related. The empirical type I errors of the Wald test exceed the nominal level for the lower prevalences, reaching 12.5% when disease prevalence in subjects completing screening in the two arms are 0.01 and 0.005. Empirical type I errors of the Wald test are closer to the nominal value when 10 000 subjects are randomized to each arm and when the sensitivity of standard test is 0.75 or 0.85. Both the likelihood ratio and score tests show adequate control of the type I error in all settings considered.

**FIGURE 3:**
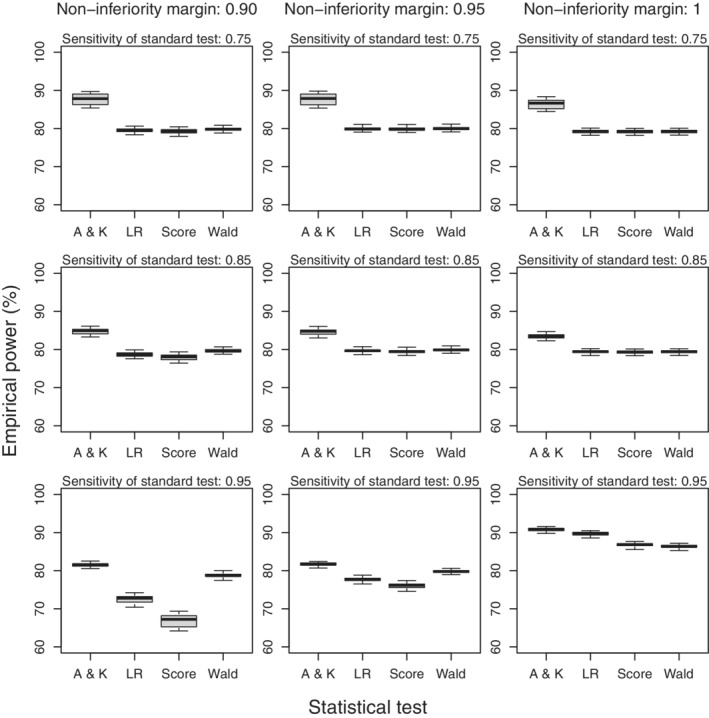
Boxplots of the empirical power of the Alonzo and Kittelson (A & K) test, likelihood ratio (LR) test, score test and Wald test in case of perfect screening uptake when the disease prevalence equals 0.01. Boxes represent quartiles and median and whiskers represent the minimum and maximum

Figure 3 shows the power of the Alonzo and Kittelson test to be higher than the power of the other three tests with absolute differences up to 7%. Both the likelihood ratio test and score test have lower power than the Wald test when the sensitivity of the standard test is 0.95 and the noninferiority margin is 0.9. This difference can at least be partly explained by the Wald test being too liberal for this combination of parameters. The difference is less pronounced for the margins of 0.95 and 1 for which the type I error for the Wald test is closer to the nominal value and required sample sizes are larger. When we set the prevalence at 0.05 and 0.10 we observed empirical powers that are similar to those observed for the prevalence of 0.01 (results not shown).

Results of the additional simulation study evaluating the estimators with respect to bias, precision and coverage probabilities of the 95% confidence intervals are given in the Supplementary Material.

## 5 EXAMPLE: THE IMPROVE STUDY

The IMPROVE study^4^ used a randomized paired screen‐positive design in which women were randomized using a 1:1 ratio to screening involving self‐collection (intervention arm) or sampling at the general practitioner (GP) (control arm). Only those women who were positive on the screening test were later retested with the other screening test and it was assessed whether they harbored CIN3+.

A total number of 187 473 women were invited of which 16 410 (8.8%) gave informed consent and were subsequently randomized. A total of 8212 (50.0%) women were randomized to self‐sampling and 8198 (50.0%) to provider‐sampling. Baseline samples were returned by 7643 (93.0%) women in the self‐sampling group compared to 6282 (76.6%) women in the provider‐sampling group. Of the samples returned by women in the self‐sampling group 569 (7.4%) tested positive. In the provider‐sampling group 451 samples (7.2%) tested positive. In the self‐sampling group a total of 73 (0.96%) of women who returned the sample were verified to have CIN3+ compared to 45 (0.72%) in the provider‐sampling group. The corresponding relative detection rate of 1.33 for self‐sampling group as compared to the provider‐sampling group may indicate a screening uptake associated difference in disease prevalence between the arms in which case the Alonzo and Kittelson estimator is biased.

Paired test results were available for 548 (96.3%) of the women who tested positive in the self‐sampling group and for 416 (92.2%) in the provider‐sampling group. Of the 548 women with paired test results in the self‐sampling group, 396 (72.3%) women were positive on the provider‐sampling test and 72 (13.1%) women were verified to have CIN3+. Of the 72 women verified to have CIN3+, 69 (95.8%) women were also positive on the provider‐sampling test. Of the 416 women with paired test results in the provider‐sampling group, 317 (76.2%) were positive on the self‐sampling test and 41 (9.9%) were verified to have CIN3+. Of the 41 women verified to have CIN3+, 39 (95.1%) women were positive on the self‐sampling test. Relative sensitivity based on the conditional estimator was 0.993. Noninferiority tests based on the conditional estimator in the group with CIN3+ using the predefined margin of 0.9 showed self‐sampling to be noninferior for detection of CIN3+ with *P*‐values of 0.038 for likelihood ratio test, 0.043 for the score‐test and 0.013 for the Wald‐test. The *P*‐value for the noninferiority test based on the Alonzo and Kittelson estimator was 0.002. Results of our simulation study suggests that the more extreme *P*‐value of the Alonzo and Kittelson test could be due to the type I error probability exceeding the nominal level when the disease prevalence is higher in the arm that receives the new screening test first.

## 6 DISCUSSION

Many new screening tests are nowadays being developed to lower the screening barrier, reduce screening‐related harms and/or reduce processing time. Participants in trials that evaluate the performance of such screening tests cannot be blinded for the screening test they receive. This may cause the screening uptake to differ between the arms with uptake likely to be associated with presence of the underlying disease. Under those circumstances, the assumptions of the standard methods for testing the relative sensitivity using the relative detection rate in unpaired screen‐positive designs and the Alonzo and Kittelson estimator for the randomized paired screen‐positive design no longer hold.

The randomized paired screen‐positive design was originally introduced as an alternative to the paired screen‐positive design in which all subjects receive both screening tests and screen‐positive subjects receive the gold standard test. Alonzo and Kittelson^3^ showed that the number of screening tests required for a randomized paired screen‐positive study is similar to that required in a paired screen‐positive study. As only a small fraction of subjects in a randomized paired screen‐positive design receives a second screening test, this means that about two times as many subjects are needed in a randomized paired screen‐positive trial in order to achieve the same power as a fully paired screen‐positive study. For studies that assess the performance of a new screening test in a population‐based screening program, accruing subjects is unlikely to be an issue and the randomized paired screen‐positive design is an efficient alternative to the fully paired screen‐positive design. The randomized paired screen‐positive design also involves lower subject burden as the majority of subjects will only receive a single screening test. However, if the costs of accruing subjects are much larger than the costs of the screening tests, a fully paired screen‐positive study is expected to be less expensive than a randomized paired screen‐positive study.

We recommend the use of the randomized paired screen‐positive design instead of a fully paired screen‐positive design in the following two situations: (i) when the first test result becomes available before the second test is performed in which case it becomes challenging to motivate subjects with a negative first test to also take the second test because the test may induce additional burden or potential harm, and (ii) when the study is also used for comparing cumulative disease risks over time of subjects negative on the new or standard screening test. Longitudinal comparisons are not possible with a fully paired screen‐positive design because all subjects with at least one positive test receive treatment. Regulatory bodies, however, often require longitudinal comparisons in addition to cross‐sectional evaluations of sensitivity and specificity, to evaluate long‐term safety. This is particularly relevant when the sensitivity is associated with disease severity because in that situation a similar sensitivity does not automatically translate into similar safety against future disease after a negative test result.

Unpaired designs are sometimes also considered for evaluating test features because they are traditionally used in the last fifth phase of the screening test evaluation. However, they do not provide a fallback for valid inference when the disease prevalence differs between arms. A possible way out is to include the randomized paired screen‐positive design in the final fifth phase of screening test evaluation by testing subjects who screen positive with the other screening test as well. In this way, differential screening uptake can still be measured while the precision of the estimated relative sensitivity of the new versus the standard test increases dramatically. If the total population is too large to conduct randomized paired screen‐positive testing, a representative subgroup may be taken instead. However, because only a small fraction of subjects in a randomized paired screen‐positive design receive a second screening test, additional costs will be small. As our method only uses screening test outcomes from subjects verified to have the disease, power of the statistical tests will be slightly lower than the power of the Alonzo and Kittelson test. Therefore, the method of Alonzo and Kittelson should still be used in screening trials in which subjects can be blinded for the test received. In other cases our method is preferable as it provides unbiased inference in a broader range of settings with only a small loss in efficiency in settings where the Alonzo and Kittelson estimator is unbiased.

## AUTHOR CONTRIBUTIONS

**Johannes Berkhof** and **Peter M. van de Ven**: Designed the study. **Andrea Bassi**: Performed the simulation study. **Peter M. van de Ven**: Drafted the manuscript. All authors revised and approved the manuscript.

## Supporting information


**Data S1** Supplementary Material

## Data Availability

The data that support the simulation findings of this study are available from the first author upon reasonable request. The data used in the example can be found in tables published in Polman et al.^4^
