# Supplementary material for: Comparing the sensitivities of two screening tests in nonblinded randomized paired screen‐positive trials with differential screening uptake
Source: Stat Med. 2021 Oct 10;40(30):6873–84. doi: 10.1002/sim.9215 (PMC9293348; doi:10.1002/sim.9215)
Supplement: Supplementary file 1 — Data S1 Supplementary Material [file SIM-40-6873-s001.pdf]

Supplementary material for 'Comparing the sensitivities of two screening tests in non-blinded randomized paired screen-positive trials with differential screening uptake'

P. M. van de Ven, A. Bassi, J.Berkhof

1 Introduction

In the main paper an alternative estimator for the relative sensitivity is derived. This supplementary material contains a similar derivation for an alternative estimator for the relative false positive fraction. In addition, details of the simulation studies are provided and results of simulation studies evaluating properties of the alternative estimator and associated 95% confidence intervals are summarized.

This supplement is organized as follows. In Section 2, the alternative estimator is derived for the relative false positive fraction. A detailed description of the simulation studies is given in Section 3. Results of simulations evaluating the Alonzo & Kittelson estimator and our alternative estimator with respect to precision, bias and the coverage probabilities and width of the 95% confidence intervals are described in Section 4

2 A conditional estimator for the relative false positive fraction

In the manuscript a conditional estimator for the relative sensitivity is derived that remains unbiased under differential screening uptake. A conditional estimator for the relative false positive fraction can be derived in a similar manner. Employing Bayes' theorem it follows that

$$P_A\left(A+\right) = \frac{P_A\left(A+, B+\right)}{P_A\left(B+ \mid A+\right)}$$

and

$$P_B\left(B+\right) = \frac{P_B\left(A+, B+\right)}{P_B\left(A+ \mid B+\right)}.$$

Conditioning these probabilities on $D-$ gives

$$P_A\left(A+ \mid D-\right) = \frac{P_A\left(A+, B+ \mid D-\right)}{P_A\left(B+ \mid A+, D-\right)}$$

and

$$P_B\left(B+ \mid D-\right) = \frac{P_B\left(A+, B+ \mid D-\right)}{P_B\left(A+ \mid B+, D-\right)}.$$

We assume that the false positive fraction of screening test A in subjects receiving test A equals the false positive fraction of screening test A when administered to the whole study population. Thus, $P_A(A+ \mid D-) = P(A+ \mid D-)$. Similarly, we assume $P_B(B+ \mid D-) = P(B+ \mid D-)$. In addition, we assume that $P_A(A+, B+ \mid D-) = P_B(A+, B+ \mid D-)$, which means that the probability of being positive on both screening tests for the subjects without the disease does not depend on the order of the tests. Then the relative false positive fraction equals

$$\Delta^{FPF} = \frac{P(A+ \mid D-)}{P(B+ \mid D-)} = \frac{P_B(A+ \mid B+, D-)}{P_A(B+ \mid A+, D-)}.$$

From this we derive a conditional estimator for the relative sensitivity as

$$\widehat{\Delta}_C^{FPF} = \frac{N^B_{\bar{D}AB}/N^B_{\bar{D}B}}{N^A_{\bar{D}AB}/N^A_{\bar{D}A}}.$$

where $N^B_{\bar{D}B} = N^B_{\bar{D}AB} + N^B_{\bar{D}\bar{A}B}$ and $N^A_{\bar{D}A} = N^A_{\bar{D}AB} + N^A_{\bar{D}A\bar{B}}$.

3 Simulation studies

Three simulation studies are performed. In the first simulation study, the empirical type I errors of the Alonzo & Kittelson test and the Wald, likelihood ratio and score test based on the conditional estimator are evaluated in settings with perfect screening uptake and settings with imperfect screening uptake. Details of the first simulation study are given in Section 3.1. In the second simulation study, the power of the Alonzo & Kittelson test and the Wald, likelihood ratio and score test based on the conditional estimator are evaluated in the setting with perfect screening uptake. Details of the second simulation study are given in Section 3.2. In the third simulation study, we evaluate the bias and mean squared error (MSE) of the Alonzo & Kittelson estimator and the conditional estimator for the relative sensitivity and the width and coverage of the 95% confidence interval for the relative sensitivity based on the Alonzo & Kittelson test and those based on our Wald, likelihood ratio and score test. In the third simulation study, we consider both settings with perfect screening uptake and settings with imperfect screening uptake. Details of the third simulation study are given in Section 3.3. All simulations are performed in R.

3.1 Simulation study for evaluation of empirical type I error probability

Separate simulations are performed for the settings with perfect and imperfect screening uptake. In both settings, the numbers of subjects M_A and M_B randomized to each arm are assumed equal and set at 5,000 and 10,000.

In settings with perfect screening uptake, 0% withdrawal is assumed for both arms. The number of subjects actually screened is correspondingly set at $N^A = M^A$ and $N^B = M^B$. The disease prevalences $P_A(D+)$ and $P_B(D+)$ in the subjects actually screened in the two arms are assumed equal and set at 0.01, 0.05, and 0.10.

In settings with imperfect screening uptake, 0% withdrawal is assumed for the subjects randomized to the new screening test A and 20% withdrawal is assumed in subjects randomized to the standard screening test B. The number of subjects actually screened is correspondingly set at $N^A = M^A$ and $N^B = 0.8 \times M^B$. The disease prevalence $P_A(D+)$ in subjects receiving the new screening test A

as the first test is set at 0.01, 0.05, and 0.10. In the arm with the standard screening test B as first test, we set the disease prevalence $P_B(D+)$ in the subjects actually screened equal to 0.5, 0.75, 1 and 1.25 times $P_A(D+)$.

In settings with perfect and imperfect screening uptake, the sensitivity $P(B+ \mid D+)$ of standard screening test B is set at 0.75, 0.85 and 0.95. The margin δ_0 for the relative sensitivity is set at 0.90, 0.95 and 1. Sensitivity $P(A+ \mid D+)$ of the new screening test is calculated as $P(A+ \mid D+) = \delta_0 \times P(B+ \mid D+)$ to simulate data under the null hypothesis. The odds ratio OR_{sens} of new screening test A versus standard screening test B outcomes in subjects with the disease is set at 1, 2, and 5. Specificities $P(A- \mid D-)$ and $P(B- \mid D-)$ of screening test A and B are set at 0.90, 0.95 and 0.99. The odds ratio OR_{spec} of new screening test A versus standard screening test B outcomes in subjects without the disease is set at 1, 2, and 5.

For each combination of parameters settings, data for the randomized paired screen-positive design are generated according to the data generating mechanism in Appendix A. Simulation size is set at 10,000 replications per setting. In simulation runs where N^A_{DAB}, N^B_{DAB}, $N^A_{DA\bar{B}}$ and/or $N^B_{D\bar{A}B}$ are zero, we add 0.25 to each of these four counts. For each simulated dataset, the null hypothesis $\Delta \leq \delta_0$ is tested against the alternative hypothesis that $\Delta > \delta_0$ with the Alonzo & Kittelson test and the Wald, score and likelihood ratio test based on the conditional estimator using a one-sided significance level of 5%. The empirical type I error probability is calculated separately for each parameter setting as the proportion of simulation runs in which the null hypothesis is rejected.

3.2 Simulation study for evaluation of empirical power

Empirical power of the statistical tests is evaluated under the setting with perfect screening uptake. The number of subjects actually screened is correspondingly set at $N^A = M^A$ and $N^B = M^B$. The disease prevalences $P_A(D+)$ and $P_B(D+)$ in the subjects actually screened in the two arms are assumed equal and set at 0.01, 0.05, and 0.10.

Sensitivity $P(B+ \mid D+)$ of standard screening test B is set at 0.75, 0.85 and 0.95. The margin δ_0 for the relative sensitivity is set at 0.90, 0.95 and 1. The relative sensitivity δ_1 under the alternative is set at 1 for $\delta_0 = 0.90$ and $\delta_0 = 0.95$, and at 1.05 for $\delta_0 = 1$. Sensitivity $P(A+ \mid D+)$ of the new screening test is calculated as $P(A+ \mid D+) = \delta_1 \times P(B+ \mid D+)$ to simulate data under the alternative hypothesis. The odds ratio OR_{sens} of new screening test A versus standard screening test B outcomes in subjects with the disease is set at 1, 2, and 5. Specificities $P(A- \mid D-)$ and $P(B- \mid D-)$ of screening test A and B are set at 0.90, 0.95 and 0.99. The odds ratio OR_{spec} of new screening test A versus standard screening test B outcomes in subjects without the disease is set at 1, 2, and 5.

For each parameter setting, the number of subjects randomized is determined such that the power of the Wald test based on the conditional estimator equals 80%. To this end, the required number of subjects with the disease who are positive on the first screening test is calculated using the sample size formula of Farrington and Manning (1990). The total number of subjects to be randomized is subsequently derived from these numbers using the disease prevalence and sensitivity of the standard screening test. Details and formula are provided in Appendix B, where we also explain how the formula can be used to determine the trial size in settings where disease-related differential screening uptake is anticipated.

For each combination of parameter settings, data for the randomized paired screen-positive design are generated according to the data generating mechanism in Appendix A. Simulation size is set at 10,000 replications per setting. In simulation runs where N^A_{DAB}, N^B_{DAB}, $N^A_{DA\bar{B}}$ and/or $N^B_{D\bar{A}B}$

are zero, we add 0.25 to each of these four counts. For each simulated dataset, the null hypothesis $\Delta \leq \delta_0$ is tested against the alternative hypothesis that $\Delta > \delta_0$ with the Alonzo & Kittelson test and the Wald, score and likelihood ratio test based on the conditional estimator using a one-sided significance level of 5%. The empirical power is calculated separately for each parameter setting as the proportion of simulation runs in which the null hypothesis is rejected.

3.3 Simulation study for evaluating the properties of estimators and confidence intervals

Separate simulations are performed for the settings with perfect and imperfect screening uptake. In both settings, the numbers of subjects M_A and M_B randomized to each arm are assumed equal and set at 5,000 and 10,000.

In settings with perfect screening uptake, 0% withdrawal is assumed for both arms. The number of subjects actually screened is correspondingly set at $N^A = M^A$ and $N^B = M^B$. The disease prevalences $P_A(D+)$ and $P_B(D+)$ in the subjects actually screened in the two arms are assumed equal and set at 0.01, 0.05, and 0.10.

In settings with imperfect screening uptake, withdrawal is assumed 0% for the subjects randomized to the new screening test A and 20% withdrawal is assumed in subjects randomized to the standard screening test B. The number of subjects actually screened is correspondingly set at $N^A = M^A$ and $N^B = 0.8 \times M^B$. The disease prevalence $P_A(D+)$ in subjects receiving the new test A as the first test is set at 0.01, 0.05, and 0.10. In the arm with standard screening test B as first test, we set the disease prevalence $P_B(D+)$ in the subjects actually screened equal to 0.5, 0.75, 1 and 1.25 times $P_A(D+)$.

In settings with perfect and imperfect screening uptake, the sensitivity $P(B+ \mid D+)$ of standard screening test B is set at 0.75, 0.85 and 0.95. The relative sensitivity Δ is set at 0.90, 0.95, 1 and 1.05. Sensitivity $P(A+ \mid D+)$ of the new screening test is calculated as $P(A+ \mid D+) = \Delta \times P(B+ \mid D+)$. The odds ratio OR_{sens} of new screening test A versus standard screening test B outcomes in subjects with the disease is set at 1, 2, and 5. Specificities $P(A- \mid D-)$ and $P(B- \mid D-)$ of screening test A and B are set at 0.90, 0.95 and 0.99. The odds ratio OR_{spec} of new screening test A versus standard screening test B outcomes in subjects without the disease is set at 1, 2, and 5.

For each combination of parameters settings, data for the randomized paired screen-positive design are generated according to the data generating mechanism in Appendix A. Simulation size is set at 10,000 replications per setting. In simulation runs where N^A_{DAB}, N^B_{DAB}, $N^A_{DA\bar{B}}$ and/or $N^B_{D\bar{A}B}$ are zero, we add 0.25 to each of these four counts. For each dataset simulated, the Alonzo & Kittelson estimator and conditional estimator are calculated. The 95% confidence interval for the Alonzo & Kittelson estimator is calculated using the formula derived in Alonzo and Kittelson (2006). For the conditional estimator, three different 95% confidence intervals are calculated: a Wald confidence interval and intervals based on the likelihood ratio and score test. The Wald confidence interval is computed using the normal approximation for the logarithm of $\widehat{\Delta}_C = \frac{N^B_{DAB}/N^B_{DB}}{N^A_{DAB}/N^A_{DA}}$ with variance estimated by

$$\widehat{\mathrm{Var}}\left(\widehat{\Delta}_C\right) = \frac{1}{N^B_{DAB}} + \frac{1}{N^A_{DAB}} - \frac{1}{N^B_{DB}} - \frac{1}{N^A_{DA}}$$

as given in Altman (1991). The 95% confidence intervals based on the likelihood ratio and score test are calculated as the range of values for δ_0 for which the null hypothesis $\Delta = \delta_0$ is not rejected in favor of the two-sided alternative that $\Delta \neq \delta_0$ assuming a two-sided significance level of 5%. An

iterative procedure is used to find this range.

Mean bias and mean squared error are calculated for the two estimators separately for each parameter setting. Estimators are further compared using the Pitman closeness criterion by calculating the proportion of runs in which the Alonzo & Kittelson estimator was closer to the true relative sensitivity. For each parameter setting, coverage probabilities of the 95% confidence intervals are calculated as the proportion of simulation runs in which the true relative sensitivity Δ is included in the confidence interval. In addition, the average width of the 95% confidence intervals is calculated.

4 Simulation results for evaluation of estimators and confidence intervals

Figure 1 shows the coverage probabilities of the four different 95% confidence intervals for the setting with perfect screening uptake where disease prevalence is 0.01 and the sensitivity of the standard screening tests is 0.95. In this setting, most coverage probabilities are around or above the nominal value of 95%. Only when the relative sensitivity is 1.05, coverage probabilities of the Alonzo and Kittelson confidence interval and the Wald confidence interval based on the conditional estimator are below the nominal level, reaching values as low as 91%. In settings with higher prevalence or lower sensitivity, coverage was found to be close to the nominal value of 95% for all four confidence intervals. In settings with perfect screening uptake, we found confidence intervals based on the conditional estimator to be wider than the 95% confidence interval based on the Alonzo & Kittelson estimator. The 95% confidence intervals based on the conditional estimator were on average 13% (likelihood ratio based), 16% (score based interval) and 10% (Wald) wider than the 95% confidence intervals based on the Alonzo & Kittelson estimator. Comparing the Alonzo & Kittelson estimator and the conditional estimator in settings with perfect uptake using Pitman's closeness criterion, the Alonzo & Kittelson estimator was found to be closer to the true relative sensitivity in 56.4% of all simulation runs. On average mean squared errors were found to be 19% higher for the conditional estimator in settings with perfect screening uptake.

Figure 2 shows the bias of the Alonzo & Kittelson estimator and the conditional estimator for the setting with 20% withdrawal in subjects randomized to the standard screening test first and where 5,000 subjects are randomized to each arm and disease prevalence in subjects randomized to receive the new test first is 0.01. The Alonzo & Kittelson estimator for the relative sensitivity is severely biased when screening uptake is differential and related to the disease. Bias is larger when sensitivities are lower and ratio of disease prevalences is further away from 1. The conditional estimator remains unbiased in all settings. Figure 3 shows the coverage probabilities of the four different confidence intervals for the setting with 20% withdrawal in subjects randomized to the standard screening test first and where 5,000 subjects are randomized to each arm and sensitivity of the standard test is 0.95. Coverage probabilities of the Alonzo & Kittelson confidence interval are not controlled when screening uptake is differential and disease-related. Coverage probabilities below the nominal level are also observed for the Wald confidence interval based on the conditional estimator when disease prevalences are low. Coverage probabilities for the Wald confidence interval based on the conditional estimator are closer to the nominal value when $10,000$ subjects are randomized to each arm and when the sensitivity of standard test is 0.75 or 0.85. Both the likelihood ratio based and score based confidence intervals show adequate coverage in all settings considered.

References

Alonzo TA, Kittelson JM. A novel design for estimating relative accuracy of screening tests when complete disease verification is not feasible. *Biometrics.* 2006;62:605–612.

Altman DG. *Practical statistics for medical research.* London: Chapman and Hall.

Farrington CP, Manning G. Test statistics and sample size formulae for comparative binomial trials with null hypothesis of non-zero risk difference or non-unity relative risk. *Statistics in Medicine.* 1990;9:1447–1454.

Appendix A Data generating mechanism

The following procedure was used for simulating the datasets.

1. The probabilities $P(A+, B+ \mid D+)$, $P(A-, B+ \mid D+)$, $P(A+, B- \mid D+)$ and $P(A-, B- \mid D+)$ for each combination of screening outcomes in subjects with the disease are calculated from the sensitivities $P(A+ \mid D+)$ and $P(B+ \mid D+)$ and odds ratio OR_{sens}.
2. Conditional probabilities for being positive on the second screening test in subjects with the disease and positive on the first screening test are calculated as
   - $P_A(B+ \mid A+, D+) = P(A+, B+ \mid D+) / P(A+ \mid D+)$
   - $P_B(A+ \mid B+, D+) = P(A+, B+ \mid D+) / P(B+ \mid D+)$
3. The probabilities $P(A+, B+ \mid D-)$, $P(A-, B+ \mid D-)$, $P(A+, B- \mid D-)$ and $P(A-, B- \mid D-)$ for each combination of screening outcomes in subjects without the disease are calculated from the false positive fractions $P(A+ \mid D-)$ and $P(B+ \mid D-)$ and odds ratio OR_{spec}.
4. Conditional probabilities for being positive on the second screening test in subjects without the disease and positive on the first screening test are calculated as
   - $P_A(B+ \mid A+, D-) = P(A+, B+ \mid D-) / P(A+ \mid D-)$
   - $P_B(A+ \mid B+, D-) = P(A+, B+ \mid D-) / P(B+ \mid D-)$
5. For the trial arm randomized to receive the new screening test A first, data is generated as follows:
   (a) The number of subjects N_D^A who complete screening and have the disease is drawn from a binomial distribution with parameters N^A and $P_A(D+)$.
   (b) The number of subjects $N_{\bar{D}}^A$ who complete screening and do not have the disease is calculated as $N_{\bar{D}}^A = N^A - N_D^A$.
   (c) The number of subjects N_{DA}^A who complete screening, have the disease and are positive on the new screening test A is drawn from a binomial distribution with parameters N_D^A and $P(A+ \mid D+)$.
   (d) The number of subjects $N_{D\bar{A}}^A$ who complete screening, have the disease and are negative on the new screening test A is calculated as $N_{D\bar{A}}^A = N_D^A - N_{DA}^A$.

(e) The number of subjects N^A_{DAB} who complete screening, have the disease and are positive on both screening tests is drawn from a binomial distribution with parameters N^A_{DA} and $P_A(B+|A+, D+)$.

(f) The number of subjects $N^A_{DA\bar{B}}$ who complete screening, have the disease and are positive on the new screening test A and negative on the standard screening test B is calculated as $N^A_{DA\bar{B}} = N^A_{DA} - N^A_{DAB}$.

(g) The number of subjects $N^A_{\bar{D}A}$ who complete screening, do not have the disease and are positive on the new screening test A is drawn from a binomial distribution with parameters $N^A_{\bar{D}}$ and $P(A+ \mid D-)$.

(h) The number of subjects $N^A_{\bar{D}\bar{A}}$ who complete screening, do not have the disease and are negative on the new screening test A is calculated as $N^A_{\bar{D}\bar{A}} = N^A_{\bar{D}} - N^A_{\bar{D}A}$.

(i) The total number of subjects $N^A_{\bar{A}}$ who complete screening and are negative on the new screening test A is calculated as $N^A_{\bar{A}} = N^A_{D\bar{A}} + N^A_{\bar{D}\bar{A}}$.

(j) The number of subjects $N^A_{\bar{D}AB}$ who complete screening, do not have the disease and are positive on both screening tests is drawn from a binomial distribution with parameters $N^A_{\bar{D}A}$ and $P_A(B+|A+, D-)$.

(k) The number of subjects $N^A_{\bar{D}A\bar{B}}$ that complete screening, do not have the disease and are positive on the new screening test A and negative on the standard screening test B is calculated as $N^A_{\bar{D}A\bar{B}} = N^A_{\bar{D}A} - N^A_{\bar{D}AB}$.

(l) In this arm, we observe numbers $N^A_{DAB}, N^A_{DA\bar{B}}, N^A_{\bar{D}AB}, N^A_{\bar{D}A\bar{B}}$ and $N^A_{\bar{A}}$.

6. For the trial arm randomized to receive the standard screening test B first, data is generated as follows:

(a) The number of subjects N^B_D who complete screening and have the disease is drawn from a binomial distribution with parameters N^B and $P_B(D+)$.

(b) The number of subjects $N^B_{\bar{D}}$ who complete screening and do not have the disease is calculated as $N^B_{\bar{D}} = N^B - N^B_D$.

(c) The number of subjects N^B_{DB} who complete screening, have the disease and are positive on the new screening test B is drawn from a binomial distribution with parameters N^B_D and $P(B+ \mid D+)$.

(d) The number of subjects $N^B_{D\bar{B}}$ who complete screening, have the disease and are negative on the new screening test B is calculated as $N^B_{D\bar{B}} = N^B_D - N^B_{DB}$.

(e) The number of subjects N^B_{DAB} who complete screening, have the disease and are positive on both screening tests is drawn from a binomial distribution with parameters N^B_{DB} and $P_B(A+|B+, D+)$.

(f) The number of subjects $N^B_{D\bar{A}B}$ who complete screening, have the disease and are negative on the new screening test A and positive on the standard screening test B is calculated as $N^B_{D\bar{A}B} = N^B_{DB} - N^B_{DAB}$.

(g) The number of subjects $N^B_{\bar{D}B}$ who complete screening, do not have the disease and are positive on the standard screening test B is drawn from a binomial distribution with parameters $N^B_{\bar{D}}$ and $P(B+ \mid D-)$.

(h) The number of subjects $N^B_{\bar{D}\bar{B}}$ who complete screening, do not have the disease and are negative on the standard screening test B is calculated as $N^B_{\bar{D}\bar{B}} = N^B_{\bar{D}} - N^B_{\bar{D}B}$.

(i) The total number of subjects $N_{\bar{B}}^{B}$ who complete screening and are negative on the standard screening test B is calculated as $N_{\bar{B}}^{B} = N_{D\bar{B}}^{B} + N_{\bar{D}\bar{B}}^{B}$.

(j) The number of subjects $N_{\bar{D}AB}^{B}$ who complete screening, do not have the disease and are positive on both screening tests is drawn from a binomial distribution with parameters $N_{\bar{D}B}^{B}$ and $P_B\left(A+|B+,D-\right)$.

(k) The number of subjects $N_{\bar{D}\bar{A}B}^{B}$ who complete screening, do not have the disease and are positive on the standard screening test B and negative on the new screening test A is calculated as $N_{\bar{D}\bar{A}B}^{B} = N_{\bar{D}B}^{B} - N_{\bar{D}AB}^{B}$.

(l) In this arm, we observe numbers $N_{DAB}^{B}, N_{D\bar{A}B}^{B}, N_{\bar{D}AB}^{B}, N_{\bar{D}\bar{A}B}^{B}$ and $N_{\bar{B}}^{B}$.

Appendix B Sample size formula for simulations evaluating power

Farrington and Manning (1990) give a sample size formula for testing the ratio of two independent proportions in case of unequal group sizes.

We show how the sample size formula of Farrington and Manning (1990) can be used to calculate the required number of subjects with the disease who are positive on the screening test to which they are randomized. We will use that the conditional estimator

$$\widehat{\Delta}_C = \frac{N^B_{DAB}/N^B_{DB}}{N^A_{DAB}/N^A_{DA}}. \tag{1}$$

is the ratio of two independent proportions $\hat{\pi}_A = N^B_{DAB}/N^B_{DB}$ and $\hat{\pi}_B = N^A_{DAB}/N^A_{DA}$ determined in groups of size N^B_{DB} and N^A_{DA}.

Let δ_1 ($\delta_1 > \delta_0$) denote the relative sensitivity under the alternative hypothesis for which the study needs to be powered, which means that the null hypothesis $\Delta \leq \delta_0$ should be rejected with the prespecified power when $\Delta = \delta_1$. Let θ denote an a priori estimate for the ratio N^A_{DA}/N^B_{DB} expected to be observed in the trial. Let $\pi_A = P_B\left(A+ \mid B+, D+\right)$ and $\pi_B = P_A\left(B+ \mid A+, D+\right)$ denote conditional probabilities of being positive on the second screening test determined under the prespecified alternative where $P\left(A+ \mid D+\right) = \delta_1 \times P\left(B+ \mid D+\right)$ by following steps 1 and 2 in Appendix A and using a prespecified odds ratio OR_{sens}. If we assume a one-sided significance level of $\alpha \times 100\%$ and set the power under the alternative at $(1-\beta) \times 100\%$, then following Farrington and Manning (1990), the required number of subjects with the disease who receive the new screening test A as second test can be calculated as:

$$\mathcal{N}^B_{DB} = \left(Z_{(1-\alpha)} + Z_{(1-\beta)}\right)^2 \times \frac{\left(\pi_A\left(1-\pi_A\right) + \left(\delta_0^2/\theta\right)\pi_B\left(1-\pi_B\right)\right)}{\left(\pi_A - \delta_0\pi_B\right)^2}, \tag{2}$$

where $Z_{(q)}$ denotes the qth quantile of the standard normal distribution. The required number of subjects with the disease who receive the standard screening test B as second test then follows as $\mathcal{N}^A_{DA} = \theta \times \mathcal{N}^B_{DB}$.

The use of the formula is first illustrated for the setting with perfect screening uptake, where withdrawal is 0% in both arms and it can be assumed that $P_A\left(D+\right) = P_B\left(D+\right) = P\left(D+\right)$. If N subjects are randomized using a 1:1 ratio, then the expected numbers of diseased subjects receiving screening test A and B as second test equal $\mathrm{E}\left(N^B_{BD}\right) = \frac{N}{2}P\left(B+ \mid D+\right)P\left(D+\right)$ and $\mathrm{E}\left(N^A_{AD}\right) = \frac{N}{2}P\left(A+ \mid D+\right)P\left(D+\right)$, respectively. Hence, the relative expected number of subjects with the disease for which outcomes of the second screening test are available is $\mathrm{E}\left(N^A_{AD}\right)/\mathrm{E}\left(N^B_{BD}\right) = P\left(A+ \mid D+\right)/P\left(B+ \mid D+\right)$, which under the prespecified alternative equals δ_1. In the setting with perfect uptake, we therefore set θ in (2) equal to δ_1.

The number of subjects to be randomized to each arm follows from Equation (2) by dividing the right hand side by the probability that a subject has the disease and is positive on screening test B. The total number of subjects to be randomized can therefore be calculated as

$$\mathcal{M} = 2 \times \frac{\left(Z_{(1-\alpha)} + Z_{(1-\beta)}\right)^2}{P\left(D+\right)P\left(B+ \mid D+\right)} \times \frac{\left(\pi_A\left(1-\pi_A\right) + \left(\delta_0^2/\delta_1\right)\ \pi_B\left(1-\pi_B\right)\right)}{\left(\pi_A - \delta_0\pi_B\right)^2}. \tag{3}$$

In the setting with imperfect uptake, a similar procedure can be used. Let τ_A and τ_B denote the expected proportion of subjects that withdraw from the trial after being randomized to receive the

new screening test A and the standard screening test B as the first test. Let $P_A(D+)$ and $P_B(D+)$ denote the prevalence in those who complete screening in the two trial arms. It follows that under differential screening uptake

$$\frac{\mathrm{E}\left(N_{AD}^{A}\right)}{\mathrm{E}\left(N_{BD}^{B}\right)} = \frac{(1-\tau_A)\,P_A(D+)\,P(A+ \mid D+)}{(1-\tau_B)\,P_B(D+)\,P(B+ \mid D+)},$$

which can be used to determine an a priori estimate for θ to calculate $\mathcal{N}_{DB}^{B}$ using formula (3). The total number of subjects to be randomized can then be calculated as

$$\mathcal{M} = 2 \times \frac{\mathcal{N}_{DB}^{B}}{(1-\tau_B)\,P_B(D+)\,P(B+ \mid D+)}.$$

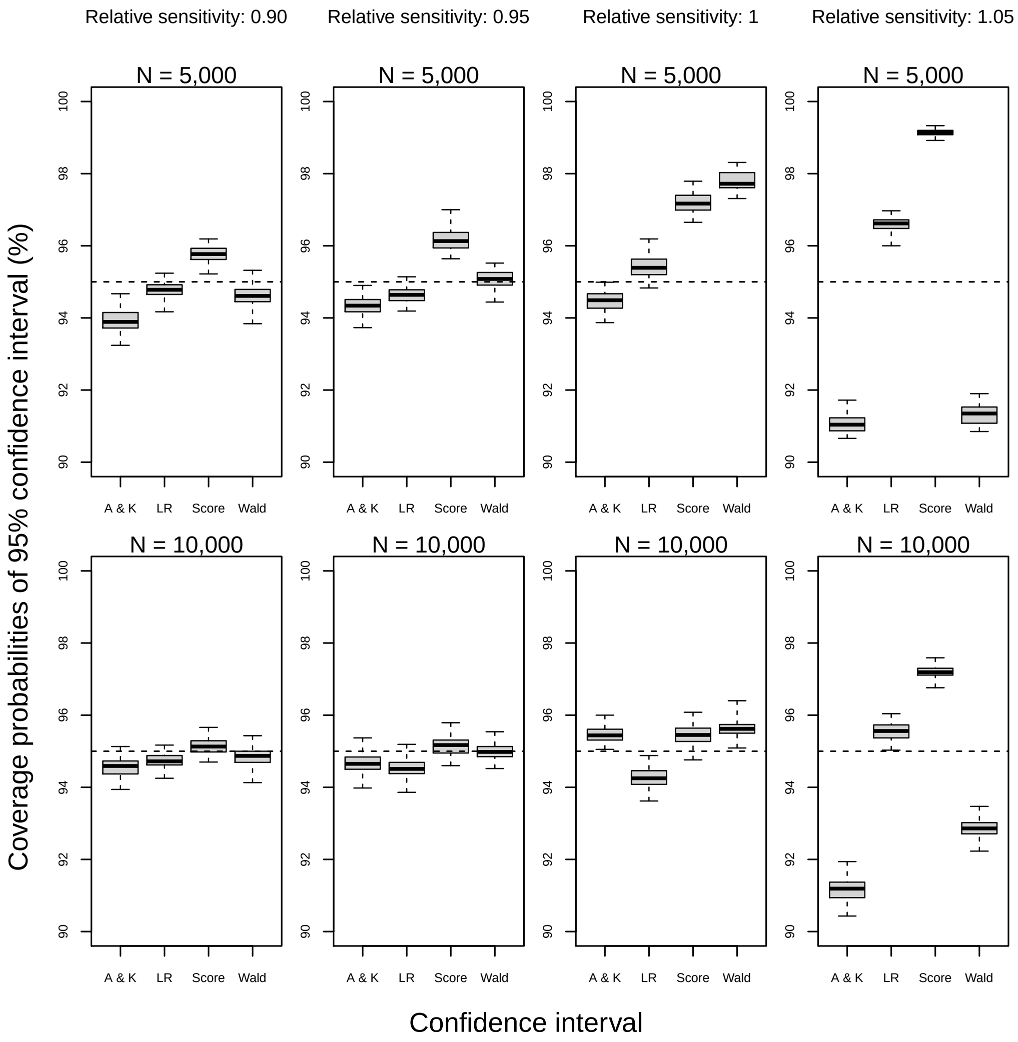

Figure 1: Boxplots of the coverage probabilities of the 95% confidence interval for the relative sensitivity of Alonzo & Kittelson (A & K) and the likelihood ratio (LR), score and Wald confidence intervals based on the conditional estimator in setting with perfect screening uptake. Disease prevalence is 0.01 and the sensitivity of the standard screening test is 0.95. Boxes represent quartiles and median and whiskers represent the minimum and maximum.

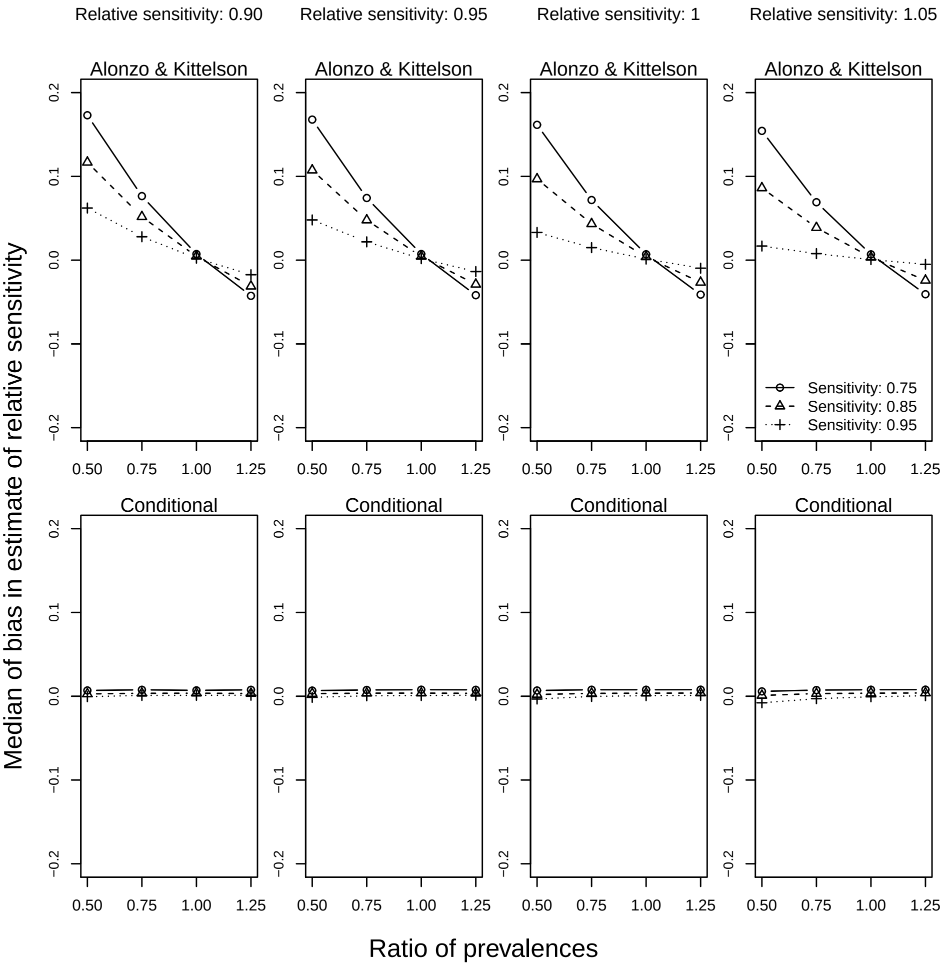

Figure 2: Median bias of the Alonzo & Kittelson and conditional estimator in setting with 20% no show in the arm receiving the standard screening test first. Perfect screening uptake is assumed for the arm receiving the new screening test first. Ratios of prevalences refer to the disease prevalence in the subjects actually screened in the arm receiving the standard test first relative to the disease prevalence in the arm receiving the new test first. Sensitivities 0.75, 0.85 and 0.95 refer to sensitivities of the standard screening test. 5,000 subjects are randomized to each arm and the disease prevalence in the arm randomized to receive the new test first is set at 0.01.

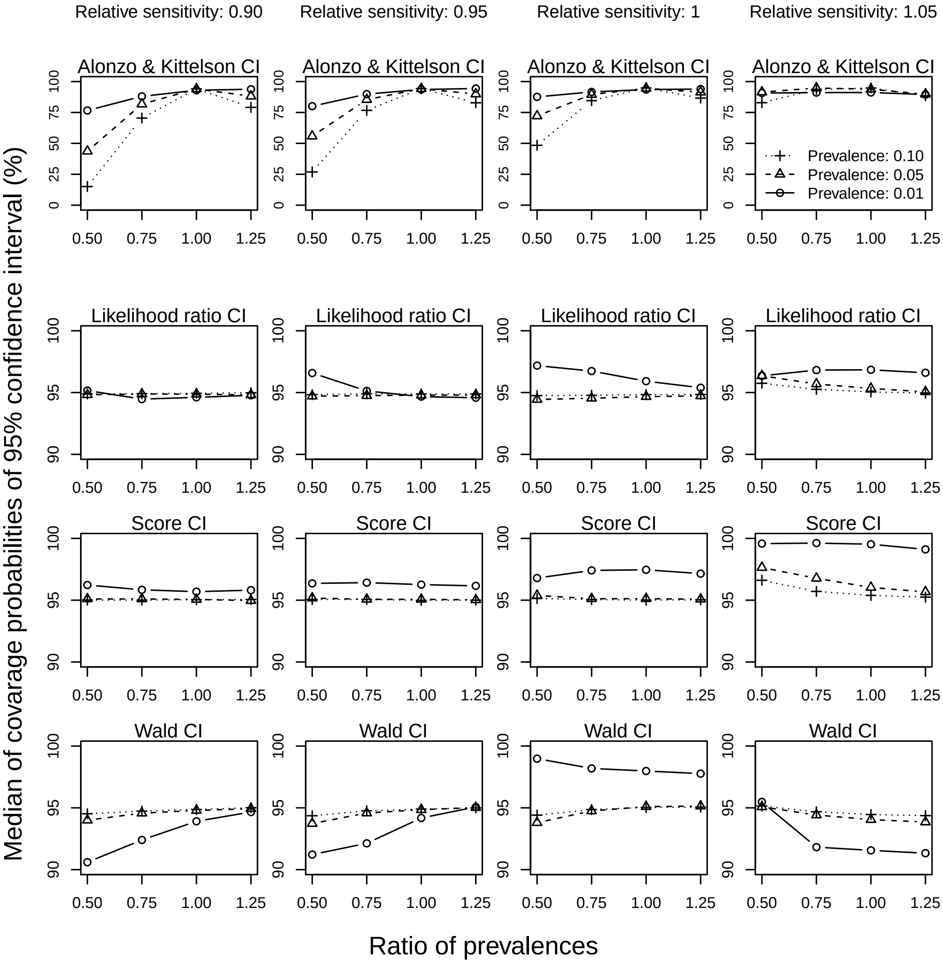

Figure 3: Median coverage probabilities of the four 95% confidence intervals in setting with 20% no show in the arm receiving the standard screening test first. Perfect screening uptake is assumed for the arm receiving the new screening test first. Ratios of prevalences refer to the disease prevalence in the subjects actually screened in the arm receiving the standard test first relative to the disease prevalence in the arm receiving the new test first. Prevalences 0.01, 0.05 and 0.10 refer to disease prevalences in the arm receiving the new test first. 5,000 subjects are randomized to each arm and the sensitivity of standard screening test is set at 0.95.
